# Localized Surface Plasmon Resonance-Based Nanosensor for Rapid Detection of Glyphosate in Food Samples

**DOI:** 10.3390/bios13050512

**Published:** 2023-04-30

**Authors:** Ariany Soares Côco, Fabiana Vasconcelos Campos, Camilo Arturo Rodríguez Díaz, Marco César Cunegundes Guimarães, Adilson Ribeiro Prado, Jairo Pinto de Oliveira

**Affiliations:** 1 Functional Nanomaterials Laboratory, Morphology Department, Federal University of Espírito Santo (UFES), Av Marechal Campos 1468, Vitória 29040-090, ES, Brazil; 2 Telecommunications Laboratory, Electrical Engineering Department, Federal University of Espírito Santo (UFES), Av Fernando Ferrari 514, Vitória 29075-910, ES, Brazil; 3 Federal Institute of Espírito Santo (IFES), km 6.5 ES 010, Vitória 29173-087, ES, Brazil

**Keywords:** biosensing, glyphosate, gold nanoparticles, localized surface plasmon resonance, cysteamine, anti-glyphosate antibody

## Abstract

In this study, we developed a biosensor based on the localized surface plasmon resonance (LSPR) phenomenon of gold nanoparticles (AuNPs) to detect the widely used herbicide glyphosate in food samples. To do so, either cysteamine or a specific antibody for glyphosate were conjugated to the surface of the nanoparticles. AuNPs were synthesized using the sodium citrate reduction method and had their concentration determined via inductively plasma coupled mass spectrometry. Their optical properties were analyzed using UV-vis spectroscopy, X-ray diffraction, and transmission electron microscopy. Functionalized AuNPs were further characterized via Fourier-transform infrared spectroscopy, Raman scattering, Zeta potential, and dynamic light scattering. Both conjugates succeeded in detecting the presence of glyphosate in the colloid, although nanoparticles functionalized with cysteamine tended to aggregate at high concentrations of the herbicide. On the other hand, AuNPs functionalized with anti-glyphosate functioned at a broad concentration range and successfully identified the presence of the herbicide in non-organic coffee samples and when it was added to an organic coffee sample. This study demonstrates the potential of AuNP-based biosensors to detect glyphosate in food samples. The low-cost and specificity of these biosensors make them a viable alternative to current methods for detecting glyphosate in foodstuffs.

## 1. Introduction

Glyphosate [*N*-(phosphonomethyl) glycine] is a broad-spectrum, post-emergent herbicide used to control weeds in agricultural crops [1]. This organophosphorus compound acts by inhibiting the 5-enolpyruvyl-shikimate-3-phosphate synthase enzyme, which is involved in the metabolism of aromatic amino acids in plants [2]. Glyphosate is present as an active ingredient in commercial formulations, such as Roundup^®^, which are popular in the agricultural community [3,4].

Currently, the widespread use of glyphosate and the perception that it is fairly safe [5] has strengthened commercialization on a large scale. However, an increasing number of studies report that it can impact the environment and human health [4,6,7,8]. For instance, glyphosate can accumulate in soil and water bodies and, when applied excessively, can affect the crops themselves [9]. Human exposure to high doses of glyphosate can lead to a range of adverse effects, including neurotoxicity and potential carcinogenicity [10]. Despite these risks, this herbicide continues to be widely used since ecological commercial alternatives are limited. Therefore, the monitoring of glyphosate is essential to protect the environment and safeguard the health of human beings [10,11,12,13,14,15].

At present, most methods employed to detect the presence of glyphosate in foodstuffs, such as liquid chromatography coupled to tandem mass spectrometry and capillary electrophoresis, although quite efficient, can be costly and require skilled personnel [16]. As efficiency goes, platforms based on nanosensors are rather versatile alternatives to the usual glyphosate detection methods, with detection limits as low as 3.4 ng·mL^−1^ having been reported for a fluorescent sensor that coupled gold nanoclusters to silica-coated carbon dots [17]. Furthermore, sensors based on the localized surface plasmon resonance (LSPR) phenomenon displayed by nanostructures such as metallic nanoparticles emerge as viable, low-cost alternatives to further approach this issue [18,19,20,21,22,23]. For instance, gold nanoparticles (AuNPs) functionalized with specific antibodies have been successfully used to detect picomolar levels of ochratoxin A—a highly toxic mycotoxin that contaminates agricultural products [24]. Likewise, trace amounts of the dithiocarbamate fungicide Ziram could be detected in water and food samples through the analysis of the LSPR property of AuNPs [25]. More to the point, a few AuNP-based biosensors that detect glyphosate in various samples have been developed recently, although sensitivity and feasibility vary considerably [26,27,28]. Different detection methods, the functionalization of AuNPs with different compounds, and the type of sample itself may account for the variation in sensitivity reported in the literature.

**Figure 1:**
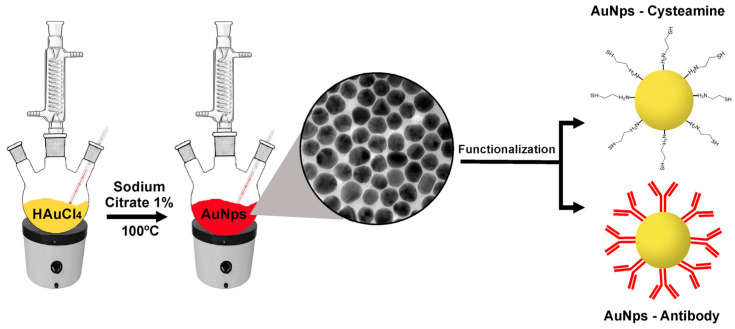
Schematic illustration depicting glyphosate detection strategies based on the functionalization of gold nanoparticles (AuNPs)—synthesized using the sodium citrate method—with either cysteamine or anti-glyphosate antibody.

In the present study, we developed an LSPR-based biosensor to detect glyphosate in food samples. For this purpose, two different detection techniques were compared: the first one was based on the functionalization of AuNPs with the mercaptoethylamine compound cysteamine, while the second technique relied on the use of a specific antibody for glyphosate conjugated to the surface of the nanoparticles (Figure 1).

On the one hand, cysteamine—a rather small molecule—should ensure optimal LSPR signals; the anti-glyphosate antibody, on the other hand, has specificity as a major strength, although signals might not be as strong. The detection strategies presented here could be an efficient alternative not only for the herbicide glyphosate, but also for other types of herbicides and pesticides based on the adaptation of the sensor with specific ligands for a given analyte.

## 2. Materials and Methods

### 2.1. Materials

All reagents used were of analytical grade. Tetrachloroauric acid (HAuCl_4_; 520918), trisodium citrate (Na_3_C_6_H_5_O_7_; S4641), cysteamine (30070), and glyphosate (2045054) were purchased from Sigma Aldrich (St. Louis, MO, USA). The polyclonal chicken anti-glyphosate IgY antibody (MBS5306191) was purchased from MyBioSource (San Diego, CA, USA). Organic and non-organic coffee grains (*Coffea arabica*) were acquired at a local market. All glassware was washed beforehand with a HNO_3_:HCl solution (1:3) and rinsed thrice with ultrapure water.

### 2.2. Instruments and Software

Ultrapure water for all preparations and tests was obtained using a Milli-Q 18Ω system (Merck-Millipore, Darmstadt, Germany). The concentration of gold nanoparticles (AuNPs) was determined using inductively coupled plasma mass spectrometry (Perkin Elmer Optima 7000; Waltham, MA, USA). The optical properties of AuNPs were evaluated using UV-vis absorption spectroscopy at 0.1 nm resolution (Ocean Optics USB 2000; Dunedin, FL, USA). The size and shape of the nanoparticles were analyzed using transmission electron microscopy (TEM-JEM-1400 JEOL microscope operated at 120 kV; Peabody, MA, USA) using the Image J software (version 1.53t, free license). Crystallinity was analyzed via X-ray diffraction (XRD) using a Philips PW 1710 diffractometer (PANalytical, Almelo, The Netherlands). Stability (Zeta potential) and hydrodynamic size were evaluated via dynamic light scattering (DLS) using a Litesizer 500 particle analyzer (Anton Paar, Graz, Austria). AuNPs were further characterized through Fourier-transform infrared spectroscopy (FTIR) using a Cary 630 spectrometer (Agilent; Santa Clara, CA, USA) and via Raman scattering using a Metrohm Instant Raman Analyzer—MIRA DS (Metrohm; Herisau, Switzerland). All plots were generated using the GraphPad Prism software, version 9.0.1.

### 2.3. Synthesis and Characterization of Gold Nanoparticles

Gold nanoparticles (AuNPs) were synthesized using the sodium citrate reduction method [29,30], in which a 1% Na_3_C_6_H_5_O_7_ solution is stirred into a boiling 2.5 × 10^−4^ M HAuCl4 solution for 15 min. The colloid was cooled in an ice bath and centrifuged at 14,000 rpm for 20 min (MiniSpin 5418, Eppendorf; Hamburg, Germany). The supernatant was discarded and the pellet resuspended in ultrapure water to remove unreacted substances. The concentration of the resulting AuNPs was determined using inductively coupled plasma mass spectrometry. The nanoparticles were characterized using UV-vis absorption spectroscopy, TEM, XRD, DLS, FTIR, and Raman scattering.

### 2.4. Functionalization of Gold Nanoparticles with Cysteamine

To coat the surface of the nanoparticles with cysteamine, 200 µL of AuNPs was mixed with 200 µL of cysteamine (0.01–2.5 mg·L^−1^) and left to stand at 25 °C for 15 min. Functionalization was monitored with UV-vis, DLS, FTIR, and Raman measurements.

The stability of AuNPs functionalized with cysteamine (AuNP-Cys; 0.1 mg·L^−1^) was analyzed using the flocculation parameter, which describes a semi-quantitative measure of aggregation using the entire absorption spectrum between 600 and 800 nm. Changes in colloid UV-vis absorption were evaluated at different ranges of pH (4–10; titrated with either 0.1 M NaOH or HCl) and ionic strength (NaCl; 0.0009–1 M), with AuNP-Cys (0.1 mg·L^−1^) resuspended in ultrapure water used as controls.

### 2.5. Functionalization of Gold Nanoparticles with Anti-Glyphosate Antibody

AuNPs were conjugated to polyclonal chicken anti-glyphosate IgY antibodies through direct electrostatic attraction. The nanoparticles were washed and resuspended in ultrapure water at pH 8, which falls near the pI of IgY proteins. Next, 6.25 µg·mL^−1^ of anti-glyphosate antibody was added to 65 µL of AuNPs and kept under orbital shaking at 150 rpm and 25 °C for 15 min. Conjugation efficiency was monitored using UV-vis, Raman, and FTIR measurements.

The stability of the AuNPs functionalized with anti-glyphosate antibody was assessed through the Gold Number assay, which finds the minimal concentration of antibody that prevents salt-induced aggregation of the nanoparticles. To do so, we prepared a serial dilution of anti-glyphosate antibody (25–0.19 µg·mL^−1^) in ultrapure water at different pH values (7–9), to which AuNPs had been previously added. Conjugation took place under orbital shaking at 150 rpm and 25 °C for 15 min. The effect of the addition of 10 µL of a 10% NaCl solution to the conjugates was then evaluated using UV-vis spectrometry.

### 2.6. Glyphosate Detection Using LSPR

The performance of AuNPs functionalized with either cysteamine or anti-glyphosate antibody in detecting glyphosate was assessed through the displacement of the LSPR band via UV-vis spectroscopy. Glyphosate concentrations varied from 0.18 to 3 mg·L^−1^ in the AuNP-Cys assay and from 0.3 to 20,000 µg·L^−1^ in the AuNP-anti-glyphosate assay. Conjugated AuNPs in the absence of glyphosate were used as controls. Ranges of detection were determined using the linear regression of the absorbance at 600 nm versus glyphosate concentration plots. Limits of detection (LOD) were determined from the linear regressions.

To evaluate the presence of glyphosate in real systems, an organic, certified coffee sample was submitted to a simple extraction phase and the sediments were diluted in 100 µL of ultrapure water. An aliquot of 0.5 µL of the herbicide (50 µg·kg^−1^) was added to 30 µL of the coffee sample and then added to 70 µL of AuNPs functionalized with the anti-glyphosate antibody. The shift in the LSPR band was then evaluated with UV-vis spectroscopy. The same procedure was adopted to assess the presence of glyphosate in a non-organic coffee sample, minus the addition of the herbicide.

## 3. Results and Discussion

### 3.1. Characterization of Gold Nanoparticles

**Figure 2:**
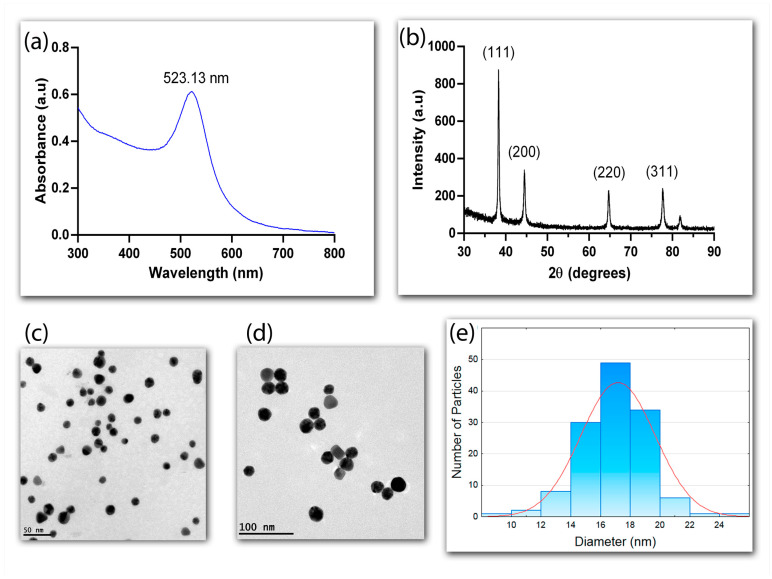
Characterization of AuNPs. UV-vis spectrum highlighting the absorption peak at 523.13 nm (**a**); X-ray diffraction pattern of AuNPs (**b**); transmission electron microscopy (TEM) images obtained using 50 nm (**c**) and 100 nm (**d**) scales; Gaussian distribution of the diameter of 500 particles obtained from TEM images (**e**).

The optical properties of AuNPs synthesized using the sodium citrate reduction method were analyzed using UV-vis spectroscopy at the 300–800 nm range (Figure 2a). The characteristic plasmonic peak at 523.13 nm confirmed the success of the synthesis. The narrow profile of the band indicated monodispersion and uniformity, due to the formation of nuclei and the increased stability provided by the citrate ions [30,31].

The crystalline nature of the AuNPs was confirmed with X-ray diffraction (XRD), which showed four diffraction peaks at 2Ө of 38.31°, 44.45°, 64.64°, and 77.73°, corresponding, respectively, to peaks (111), (200), (220), and (311) of the reflection of crystalline metallic gold (JCPDS No. 04-0784) (Figure 2b). The more pronounced diffraction peak (111) suggests that this face-centered pattern is the predominant orientation of the AuNPs.

A morphological analysis conducted through transmission electron microscopy (TEM) further attested to the uniformity and sphericity of the AuNPs (Figure 2c,d). The nanoparticles synthesized with sodium citrate were shown to be monodispersed with an approximate diameter of 17 nm (CV < 10%), according to the counting of 500 nanoparticles per sample from TEM images (Figure 2e). The concentration of the AuNPs determined using ICP-MS was 27 mg·L^−1^.

### 3.2. Functionalization of Gold Nanoparticles Using Cysteamine

**Figure 3:**
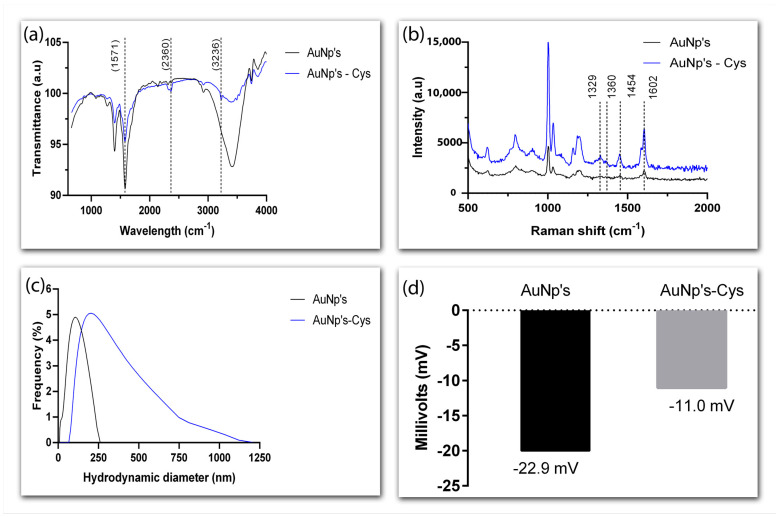
Characterization of the conjugation between AuNPs and cysteamine. Fourier-transform infrared (FTIR) spectra of naked and cysteamine-functionalized AuNPs (**a**), Raman scattering spectra of AuNPs and AuNP-Cys (**b**), dynamic light scattering (DLS) profiles of functionalized and naked AuNPs (**c**), and Zeta potential of AuNPs and AuNP-Cys (**d**).

Cysteamine has a thiol group (-SH) that forms a strong bond with AuNPs (Au-S) and an amino group (-NH2) that remains exposed on the surface of the nanoparticles. The functionalization of AuNPs with cysteamine was characterized using Fourier-transform infrared spectroscopy (FTIR), Raman scattering, Zeta potential, and dynamic light scattering (DLS) (Figure 3). FTIR spectra revealed a peak at 2360 cm^−1^ that corresponds to the elongation vibration of the cysteamine’s -SH group, while the sharp and broad peak at 1571 cm^−1^ can be attributed to the elongation of the -NH2 group. The transmittance peak at 3236 cm^−1^ is due to -NH2 stretching vibrations (Figure 3a).

Figure 3b compares the Raman spectra of AuNPs alone and AuNPs functionalized with cysteamine (AuNP-Cys). Although faint, the bands at 1329 and 1454 cm^−1^, which are characteristic of cysteamine, further confirm the success of functionalization. The weak intensity can be explained by a lack of free cysteamine in the solution. The bands at 1360 and 1602 cm^−1^ are attributed, respectively, to the symmetric and asymmetric stretching vibrations of carboxylate groups in the AuNPs [32].

To evaluate how functionalization with cysteamine changes the surface of AuNPs, the hydrodynamic size of the conjugate was analyzed using DLS (Figure 3c). It is worthy of note that the diameter of AuNPs thus analyzed was higher than the average value obtained by TEM because carboxylate ions, which influence the size of the particles, are perceptible only in the DLS analysis. Regardless, a clear increase in the hydrodynamic diameter of the nanoparticles was perceived after functionalization—188 nm as opposed to the 91 nm observed in naked nanoparticles.

The analysis of surface charge reveals the colloidal stability of the particles and whether they aggregate. Functionalization with cysteamine decreased the Zeta potential value of AuNPs from −22.9 mV to −11.0 mV (Figure 3d), reflecting the effect of the positive net charge of this mercaptomethylamine compound [33]. These results suggest functionalization with cysteamine did not affect the stability of the nanoparticles.

**Figure 4:**
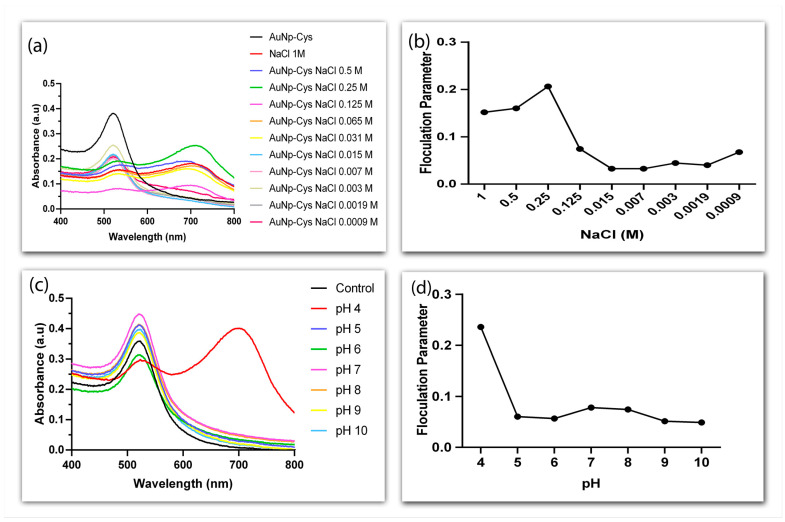
UV-vis absorption spectra of AuNPs functionalized with cysteamine at different concentrations of NaCl (0.0009–1 M) (**a**); flocculation analysis as a function of NaCl concentration in mols/L (**b**); UV-vis absorption spectra of AuNPs-Cys at control conditions (AuNPs resuspended in ultrapure water) and at pH 4 to 10 (**c**); flocculation analysis as a function of pH value (**d**).

To further assess stability, AuNP-Cys were analyzed using UV-vis spectroscopy at different NaCl concentrations and pH values (Figure 4). It was observed that functionalized AuNPs suffered aggregation from 0.031 to 1 M NaCl (Figure 4a,b). Likewise, the shifting of the plasmonic absorption peak to ~700 nm at pH 4 point to the aggregation of AuNP-Cys in these conditions. On the other hand, at pH 5 to 10 the conjugate remained stable, although slight changes in absorbance were detected at the wavelength range analyzed (Figure 4c,d).

### 3.3. Conjugation of Gold Nanoparticles with Antibodies

**Figure 5:**
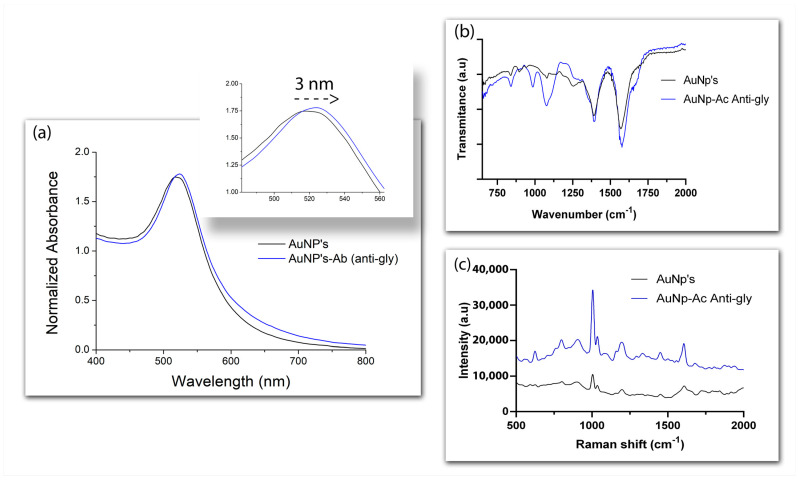
Displacement of the AuNPs’ plasmonic peak (zoomed in in the insert) in the presence of anti-glyphosate antibody (**a**). Comparison of the FTIR spectra of naked AuNPs and AuNPs conjugated to anti-glyphosate antibody (**b**). Raman spectra of naked AuNPs and AuNPs conjugated to anti-glyphosate antibody (**c**).

The physical adsorption of antibodies on the surface of nanoparticles modifies the plasmonic resonance, since the coating of the nanomaterial with a dielectric material changes the refractive index, the frequency of LSPR, and absorption [34,35,36]. To confirm the interaction between AuNPs and anti-glyphosate antibodies, which takes place through electrostatic interaction [37,38], the position of LSPR was analyzed via UV-vis spectroscopy in the presence of antibodies, using naked nanoparticles as controls (Figure 5a). The ~3 nm displacement of the plasmonic peak shown by AuNPs in the presence of the anti-glyphosate antibody confirmed the success of the conjugation. Further information on the conjugate was obtained by addressing the nature of the compound attached to the surface of the AuNPs through FTIR spectroscopy and Raman scattering. The fingerprint region near 900 cm^−1^ can be assigned to amino acid vibrations [39], while the bands detected at 991 cm^−1^ and 1100 cm^−1^ are the result of N-O stretching and NH3+ rocking vibrations, respectively [40], suggesting the anti-glyphosate antibody was indeed present in the surface of the nanoparticles (Figure 5b). Functional groups related to the interaction between AuNPs and the antibodies were also evidenced around 619 and 746 cm^−1^ in the Raman spectra, which correspond to C-S vibrations [41,42], while the bands near 1651 cm^−1^ reflect amide I vibrations (Figure 5c) [41].

### 3.4. Glyphosate Detection Using LSPR

Following the characterization of AuNPs functionalized with either cysteamine or anti-glyphosate antibodies, we moved on to compare them as to their ability to detect the presence of glyphosate, both added to the colloid and to real samples.

**Figure 6:**
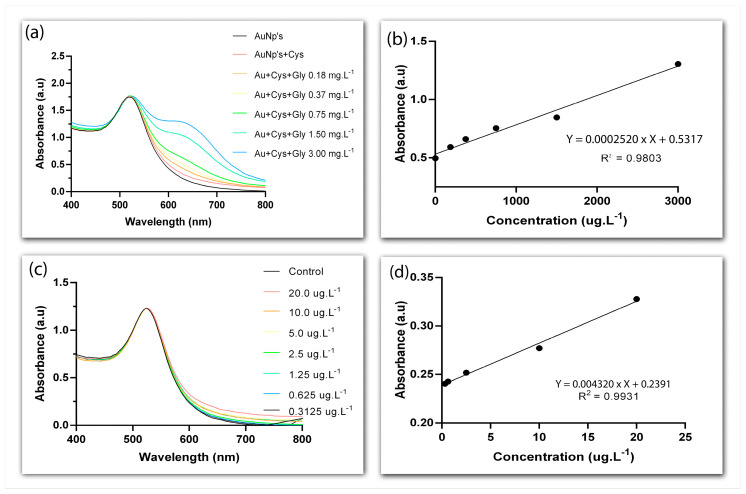
UV-vis spectra of gold nanoparticles functionalized with cysteamine in the presence of glyphosate; control curves refer to AuNP-Cys in the absence of the herbicide (**a**). Calibration curve for the detection of glyphosate with AuNP-Cys; absorbance at 600 nm (**b**). UV-vis spectra of gold nanoparticles functionalized with anti-glyphosate antibody in the presence of glyphosate; antibody-conjugated AuNPs were used as controls in the absence of glyphosate (**c**). Calibration curve for the detection of glyphosate with AuNP-anti-glyphosate antibody; absorbance at 600 nm (**d**).

The exposure of the positively charged amino group (-NH2) of cysteamine in the surface of AuNPs creates a point of interaction for the negatively charged carboxylate (-COOH) and phosphonyl groups of glyphosate (-PO_3_H_2_). This interaction was confirmed by the gradual appearance of a second LSPR band above 600 nm at increasing concentrations of the herbicide (0.18–3 mg·L^−1^), pointing to the aggregation of AuNPs (Figure 6a). It has been previously proposed that glyphosate causes AuNPs to aggregate by simultaneously cross-linking them and by decreasing surface charge density, which would upset the balance between the electrostatic and contact forces that keep nanoparticles apart [43]. Nevertheless, we attained a linear range of detection between 0.18 and 3 mg·L^−1^ (Figure 6b) and an LOD of 42 µg·L^−1^. These rather high values fall somewhat close to what has been previously reported for similar detection systems. For example, in a study in which AuNPs conjugated to cysteamine were employed to detect glyphosate in water samples through colorimetry, a linear range of ~0.084–1.183 mg·L^−1^ and an LOD of ~10 µg·L^−1^ were found [43], while yet another study employing a similar approach—although with L-cysteine instead of cysteamine—reported a linear range of ~1–23 mg·L^−1^ and an LOD of ~270 µg·L^−1^ [26].

Due to asymmetrical charge distribution, antibodies are more likely to lie flat on the surface of metallic nanoparticles when bound through physical adsorption, which should leave the antigen-binding site exposed [44]. Assuming that is the case for the anti-glyphosate antibody coating the AuNPs synthesized here, one should expect LSPR alterations in the presence of glyphosate. Indeed, we observed a plasmonic shift around 600 nm at all concentrations of glyphosate employed in this study (0.312 to 20,000 µg·L^−1^) (Figure 6c). Moreover, the higher the concentration of glyphosate, the greater the displacement in the plasmonic band, attesting to the sensitivity of the system. Linearity was detected throughout the whole glyphosate concentration range and an LOD of 0.15 µg·L^−1^ was determined (Figure 6d).

A comparison between ranges of detection and LOD values of various glyphosate detection systems, including our own, reveals that by conjugating AuNPs with specific anti-glyphosate antibodies we have achieved a high degree of sensitivity (Table 1). It is also worth mentioning that, unlike what had been observed with AuNPs-Cys, the presence of glyphosate did not cause aggregation of the antibody-functionalized AuNPs at any of the concentrations tested here. For these reasons, only AuNPs conjugated with anti-glyphosate antibodies were chosen for tests with real samples.

**Figure 7:**
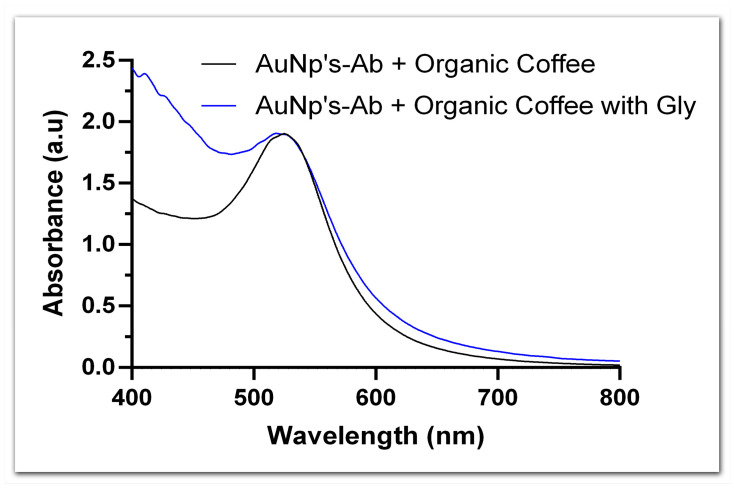
UV-vis spectra of AuNPs conjugated to anti-glyphosate antibody in the presence and absence of organic coffee samples containing glyphosate.

To verify the applicability of the detection system in real samples, a sample of organic Arabica coffee, to which glyphosate was added at a concentration of 50 µg·kg−1, was evaluated as a study model. When comparing the UV-vis spectra of AuNPs functionalized with anti-glyphosate antibody in the presence and in the absence of the glyphosate-spiked coffee sample, we detected an LSPR shift that reflected a total of 40.84 µg·L−1 of glyphosate, according to the calibration curve for this conjugate (Figure 7). Moreover, a preliminary test revealed a total of 1.17 mg·L^−1^ of glyphosate in a non-organic coffee sample, although the accuracy of this result is pending confirmation. We must, however, highlight that concentrations as high as 7.5 mg·L^−1^ have already been reported in coffee grains from the same region in which the present study took place [45], which makes our results plausible. Although preliminary, these are promising results, particularly because the detection system proposed here allows the real-time detection of glyphosate in food samples.

## 4. Conclusions

In the present study, we developed a fast, simple, and sensitive method for detecting glyphosate in food samples. Glyphosate detection was based on the LSPR phenomenon displayed by gold nanoparticles, allowing the analyte to be monitored in real time. The presence of glyphosate was confirmed by binding either cysteamine or specific antibodies to the herbicide, with a wide range of linearity in both cases. We have also shown that the LSPR system proposed here successfully identified the presence of glyphosate in real food samples, indicating that it has potential for full-scale applications, with quantification limits that cover the values allowed by national and international legislation. The system developed can be used as an alternative for rapid detection at the collection site or as a monitoring and screening tool for traditional detection systems.

## Figures and Tables

**Table 1:** Comparison between glyphosate detection systems based on nanoparticles.

Detection System	Detection Range	LOD ^1^	Reference
L-cysteine-AuNPs	1–23 mg·L^−1^	270 µg·L^−1^	[26]
Cysteamine-AgNPs	-	1700 µg·L^−1^	[27]
Cysteamine-AuNPs	0.001–1000 mg·L^−1^	-	[28]
Cysteamine-AuNPs	0.084–1.183 mg·L^−1^	10 µg·L^−1^	[43]
Cysteamine-AuNPs	0.180–3 mg·L^−1^	42 µg·L^−1^	This study
Anti-glyphosate-AuNPs	0.0003–0.02 mg·L^−1^	0.15 µg·L^−1^	This study

^1^ Limit of detection.

## Data Availability

The data presented in this study are contained within the article.
